# Dispensaries for Sailors

**Published:** 1887-08-27

**Authors:** Pietro Michelli


### DISPENSARIES FOR SAILORS.

By Pietro Michelli.

From London Bridge to the Nore is the district generally known to mariners as the Port of London, and the distance by the river is over forty miles. For the greater part of this distance on both sides of the river there are long lines of docks, wharves, roads for anchorage and all their attendant impedimenta of barges, lighters, etc., and it is estimated that every year 2,000,000 sailors enter the port and are employed on board the different vessels in dock or on the river. This is exclusive of the large class of stevedores, bargemen, and others engaged in the shipping trade. Every class of vessel finds a berth somewhere in the port, from the stately liner of 6,500 tons, floating majestically in the enormous basin of one of the docks, to the humble coaster or collier, which is often content to lie upon a mud-bank taking a short rest from the turmoil of the sea. The docks and wharves are always being extended, and now one of the largest docks is situated at Tilbury, opposite to Gravesend. Too little thought seems to be bestowed on the floating inhabitants of all these ships: to these men even the very humblest owe something for the countless daily comforts of life. Of those dwelling in London but a very small portion ever see the ships in the river, and their sympathy is not aroused for those who go to sea. Sailors, of all men, should claim our sympathy; the hardships and provocations they have to endure are unknown in almost any other vocation, while they are a class of men whose rough life seems to bring out in them many good qualities which are dormant or wanting altogether in others. It is a noteworthy fact that the sailor, who sees so much of the world under such varying aspects, should, in the popular acceptation of the term, be so little "a man of the world." To use his own terms, "when he is ashore he is adrift," so that when ill or laid up by accident, he is all the more worthy of sympathy and assistance.

This vast number of toilers look to the Seamen's Hospital Society for assistance when sick ashore; nothing can appeal more to the feelings than a sick sailor. If a landsman is ill, some one knows about him; his friends look after him, get him into a Hospital, visit him there, and take care of him during convalescence; but all this is denied the sailor; he has no such personal friend. Instead, he looks to the Seamen's Hospital Society or to the Dreadnought Hospital. Here he receives the best medical advice; is tended by skilled nurses; kept during convalescence, and when well enough, a ship is found for him, or he is sent to his home, either by the Society, or, if a foreigner, through his Consul.

The Hospital is situated in the immediate vicinity of the Surrey Commercial Docks, and within easy reach of the East and West India Docks and the shipping lying in Deptford, Greenwich, and Woolwich reaches; but, owing to the expansion of the shipping trade, the Seamen's Hospital Society has found some difficulty in collecting sick sailors from a distance. The Society has therefore found it necessary to establish Dispensaries. One has been working with much success at Well Street, London Docks, for the last five years, and in consequence of the increase in the number of sailors who now embark there, a dispensary was opened at Gravesend on the 27th July, and these dispensaries are fitted up with all the latest improvements—consulting-room, drug-store, and comfortable waiting-rooms. A physician or surgeon examines each patient, giving medicine in trivial cases and attending to slight surgical cases, while those more seriously ill who need treatment in the wards of a hospital are sent to Greenwich, in charge of experienced attendants. In this way a sailor can lie up, and be taken care of until he is fit to go afloat again. Although the dispensary at Gravesend has only been opened so short a time, there has been a great increase in the number of patients who have attended, and several have been sent to the Hospital, and are now in the wards.

The expense of maintaining one of these Dispensaries is about £300 per annum, and there is little doubt that this additional sum will be forthcoming, not only from the inhabitants of Gravesend, but also from those who have the welfare of sailors at heart. Of those who receive the benefit of the Society nearly all are friendless, and many in a strange land, and it is for the succour of these when ill that the Seamen's Hospital was established. For the last sixty-nine years the Society has diligently carried on its work, and during that time over one hundred and thirty-five thousand sailors of every nation, colour, and creed have been aided by the Hospital.

